# Partisanship overcomes framing in shaping solar geoengineering perceptions: Evidence from a conjoint experiment

**DOI:** 10.1038/s44168-025-00236-3

**Published:** 2025-03-24

**Authors:** Beatrice Magistro, Ramit Debnath, Paul O. Wennberg, R. Michael Alvarez

**Affiliations:** 1 California Institute of Technology, Pasadena, USA; 2 University of Cambridge, Cambridge, UK

**Keywords:** Social sciences, Environmental social sciences, Climate-change mitigation, Psychology and behaviour

## Abstract

The discourse on solar geoengineering (SG) is evolving, yet public perceptions of SG as a climate change solution remain underexplored, especially in the polarized US political landscape. We examine the relative importance of different SG narratives—framed as complementary, substitutive, or posing a moral hazard—and partisan information sources in shaping public attitudes. Using a conjoint experiment with 2123 American voters, we find that partisan alignment with the information source plays a decisive role in shaping trust in the messenger and support for SG, overshadowing any impact of message framing. Both Democrats and Republicans are more likely to trust the messenger and support SG when the information comes from a copartisan source. However, despite these strong partisan influences, policy preferences remain consistent with ideological baselines. These findings highlight the importance of partisanship in shaping perceptions of emerging climate technologies such as SG, even in contexts of low public awareness, and underscore the challenges of depolarizing public discourse on climate change solutions.

## Introduction

The discussion surrounding solar geoengineering (SG), also known as solar radiation management (SRM), is evolving rapidly, as these technologies gain attention for their potential role in mitigating climate change. Solar geoengineering strategies are designed to lower the temperature of the Earth through various methods, such as introducing small reflective particles into the upper atmosphere, increasing the reflective cloud cover in the lower atmosphere, or thinning high-altitude clouds that can absorb heat^1^. The 2021 National Academies of Sciences report emphasizes the urgency of addressing climate risks and recommends that the US modestly funds SG research alongside much larger investments in a wider range of climate mitigation and adaptation strategies^1^.

Despite SG’s emerging prominence in policy discussions, its potential to shape broader climate-related beliefs and behaviors remains underexplored, particularly in the context of the US’s politically polarized climate policy landscape. Given the limited familiarity of the public with SG technologies, early and effective communication will play a pivotal role in shaping perceptions of the potential benefits and risks of SG^2–7^. Research on climate change communication suggests that framing often struggles to overcome deeply embedded beliefs^8^. However, SG’s relative novelty and low public salience may create a rare window for strategic framing to meaningfully influence public opinion. Recent surveys in 22 countries support this idea, highlighting the potential of SG to redefine public discourse on climate solutions^5,9–11^.

In the United States, climate change discourse is deeply divided along partisan lines. Republicans and Democrats often have starkly different views on climate science, policy, and solutions^12,13^. Republicans tend to be more skeptical of the scientific consensus on climate change and less supportive of government regulations to address it^13–20^. This polarization complicates efforts to build consensus on climate action and raises questions about how communication on an unfamiliar issue, such as SG, might be filtered through existing ideological divides.

As discussions around SG evolve, three key perspectives have emerged. The scientific community often frames SG as a “complement” to existing climate mitigation and adaptation strategies, emphasizing its role alongside emission reductions^1^. Others frame it as a potential “substitute” for reducing greenhouse gas emissions, appealing to those who favor technological solutions over regulatory measures^21,22^. A third perspective raises concerns about the “moral hazard” SG could create by reducing the urgency of traditional climate actions^23^. Although these frames reflect different viewpoints, their influence on public perceptions is likely to depend on the broader partisan context. For example, conservatives may view a substitutionary frame as a technological fix preferable to emissions cuts^9,21,22^, while complementary frames may resonate with moderates or progressives seeking a comprehensive approach to climate solutions. However, in a polarized political landscape, the messenger’s partisan alignment may overshadow the framing itself, shaping how these perspectives are received.

This study examines the relative importance of SG message framing and the partisan identity of information sources on public trust in messengers, support for SG, and broader climate policy preferences. Using a representative sample of 2123 American registered voters, we employ a conjoint experiment to systematically vary message framing, the partisan alignment of the information source, and the source’s professional role (researcher or podcaster). This approach mirrors the complexity of real-world communication, where audiences encounter multiple, overlapping cues and frames.

Although prior research shows that partisan signals strongly influence responses to climate information^13,24–26^, it is not clear whether this pattern holds for low-salience and still relatively depoliticized technologies such as SG. By examining framing effects alongside partisan cues, we assess whether SG is susceptible to entrenched polarization or whether certain frames could mitigate or amplify partisan divides.

Our findings reveal that the partisan alignment between the information source and the respondent plays a crucial role in shaping attitudes toward SG. Republicans are more likely to trust the messenger and support SG when the information comes from a source aligned with their political identity, while Democrats show similar patterns with copartisan sources. Interestingly, framing SG as a complement, substitute, or moral hazard had minimal impact compared to the influence of the partisan messenger. These results suggest that in a politically polarized environment, the effectiveness of SG communication strategies will depend more on the perceived political alignment of the messenger than on the content of the message itself. This has significant implications for policymakers and advocates who engage the public on emerging climate technologies such as SG, particularly in contexts marked by deep ideological divides.

Public opinion on climate change in the United States is strongly shaped by partisan identities and ideological predispositions. Conservatives and Republicans tend to be more skeptical of human-caused climate change and less supportive of government interventions, whereas liberals and Democrats generally endorse the scientific consensus and favor mitigation efforts^14,16,19,20^. This partisan divide reflects deep-rooted processes of politically motivated reasoning and selective exposure to information, as people actively seek and interpret climate-related content through their ideological lenses^24,27–29^. Over time, these processes have entrenched sharp divides in public attitudes toward climate policies, complicating efforts to build consensus on climate action.

Solar geoengineering enters this polarized landscape as a relatively low-salience emerging climate technology. Unlike well-established mitigation strategies, SG has not yet become deeply politicized or widely covered by the media. This limited public awareness theoretically creates an opening for less ideologically constrained evaluations. However, the mixed evidence on low-salience issues suggests that even unfamiliar technologies may not escape existing partisan filters. Although some research shows that when people know little about an issue, they rely heavily on heuristics such as partisanship^30–32^, other studies indicate that policy content can matter more for low-salience policies^33^. In the case of SG, the broader domain of climate policy is highly salient and polarized, which may cause respondents to link SG to underlying partisan attitudes despite limited direct exposure.

How SG is presented, or framed, may also shape public perceptions. Framing involves focusing on certain aspects of an issue, influencing how people conceptualize and evaluate it^34–36^. A rich literature shows that climate attitudes can change with frames that emphasize public health and national security^37^, local impacts^38^, gains versus losses perspectives^39^, or scientific consensus^40^.

In the case of solar geoengineering, three primary frames have been identified. First, the “complementary” frame presents SG as part of a wider climate strategy, supplementing emission reductions rather than replacing them^1^. Second, the “substitution” frame suggests that SG could serve as a standalone technological fix, appealing to conservatives who prefer market-based fixes or solutions that avoid regulatory actions and emission cuts^4,21,22^. Third, the frame “moral hazard” posits that SG could reduce the urgency for traditional mitigation by offering an illusory shortcut, thus deterring efforts to reduce emissions^23,41–44^. Empirical findings on these frames are mixed: some research finds evidence of moral hazard^6^, others find no effect of information on the support of mitigation^43,45^, and still others identify a “reverse moral hazard effect ”^22,41,46–48^.

These different frames also highlight the governance challenges associated with SG research and potential deployment. Key concerns include the transboundary effects of large-scale outdoor activities and the global implications of the implementation of SG^44^. SG introduces what some see as a “techno-solutionist” approach to climate change mitigation, contrasting with environmentalist perspectives that prioritize minimal intervention in nature as a path to sustainability^49^. Additionally, SG’s benefits are often designed for a single actor, like a state, although others may disagree on whether, when, or how SG should be implemented^44^. These uncertainties make SG decisions inherently political, raising questions about international cooperation, global climatic goals, and the prioritization of climate goals relative to other national interests^23,50^. In this context, public participation is essential to shape the direction of SG research, development, and governance^44,51^.

However, framing rarely operates in a vacuum. Information about emerging technologies such as SG is conveyed through messengers embedded in partisan and ideological networks. Political elites, the media, advocacy groups, and think tanks all shape the informational environment. In the context of US climate policy, partisan alignment often dictates which sources are considered credible^52–54^. As a result, even the most carefully crafted frames may have limited efficacy if presented by a source whose partisan identity conflicts with the recipient’s own political predispositions.

Research consistently demonstrates that individuals use party cues as cognitive shortcuts, especially when faced with complex or unfamiliar issues^13,27,55^. In climate and energy policy, Republicans and Democrats respond differently to identical proposals depending on the perceived partisan affiliation of the messenger^25,26^. Republicans tend to be less trusting of scientific authorities on climate issues and more inclined to follow copartisan signals, whereas Democrats are often more responsive to scientific expertise and Democratic-aligned communicators^16,17^. This partisan divide in trust in expertise is not unique to climate change, but reflects broader ideological patterns. Conservatives are generally more skeptical of academic and scientific institutions, which they often perceive as aligned with liberal values or agendas^56^.

These insights suggest that understanding public responses to SG requires considering not only how the technology is framed but also who delivers that information. If partisan identity dominates from the outset, SG could quickly become politicized, mirroring polarization in other areas of climate policy. Conversely, if certain frames can transcend partisan boundaries—either by appealing to shared values or by presenting SG in a manner that resonates with both ideological camps—there may be opportunities to foster more constructive engagement at an early stage.

In this study, we examine the relative importance of SG message framing and the partisan identity of the messenger in shaping public attitudes. Using a conjoint experiment that varies both the frame (complement, substitute, moral hazard) and the source (Democratic or Republican, researcher, or podcaster), we simulate a realistic informational environment. Rather than isolating one factor, we assess how multiple attributes jointly influence trust in the messenger, support for SG, and preferences for mitigation strategies. Our approach acknowledges that, in the real world, audiences encounter a mix of content and signals simultaneously, and their responses emerge from the combined influence of these elements.

Drawing on the literature discussed above, we propose the following hypotheses:

- H1 (Partisan Source Effects): Individuals will trust messengers and support SG more when information is delivered by a copartisan source. Republicans will respond more favorably to sources aligned with the Republican Party, while Democrats will respond more favorably to sources aligned with the Democratic party.
- H2 (Messenger Occupation Effects): Republicans will respond more favorably to podcasters, while Democrats will respond more favorably to researchers.
- H3 (Framing Effects): Republicans are expected to be more supportive of SG in a “substitution” frame that emphasizes SG as a technological fix. Democrats, in contrast, may show stronger support under a “complementary” frame that affirms the need for emissions reductions alongside SG, compared to substitution or moral hazard frames.
- H4 (Termination Shock Information): Highlighting the abrupt warming risks of stopping SG (termination shock) should temper support for SG among individuals from both parties, but through different processes. Democrats may reaffirm the primacy of emissions reductions, while Republicans may become cautious about relying on SG alone, diminishing its appeal as a straightforward substitute.

## Results

**Fig. 1:**
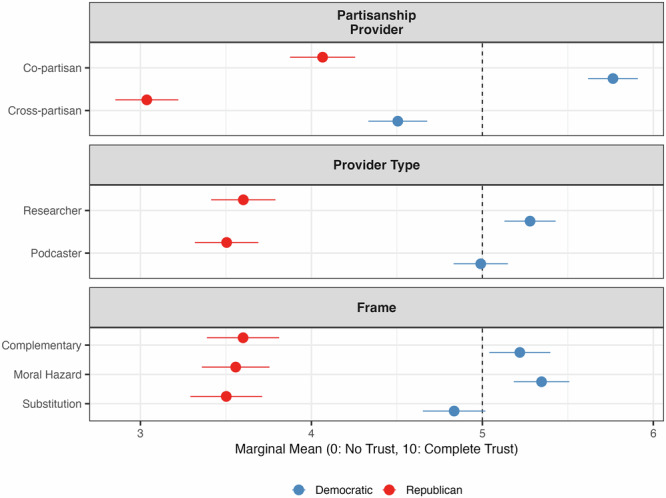
Marginal means of trust in the information source by respondent’s partisanship. Marginal means show the average outcome for each specific level of a conjoint attribute, averaging across all other attributes. Error bars represent 95% confidence intervals.

**Fig. 2:**
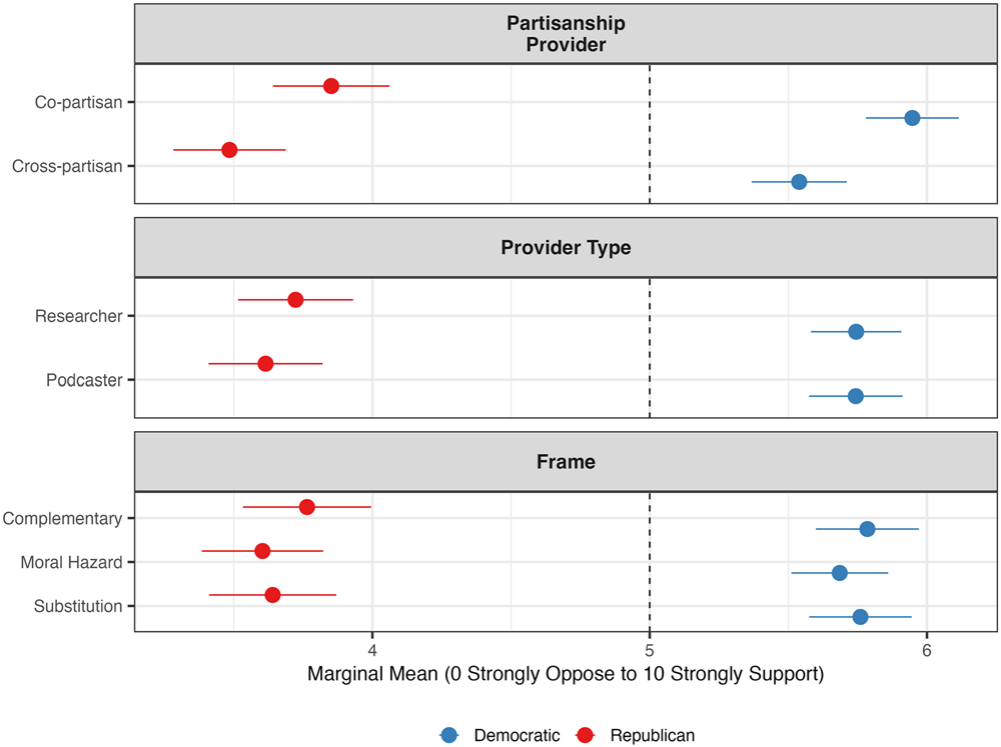
Marginal means of support for SG by respondent’s partisanship. Marginal means show the average outcome for each specific level of a conjoint attribute, averaging across all other attributes. Error bars represent 95% confidence intervals.

We begin by examining how the partisanship of information sources, their occupational roles (researchers or podcasters), and the framing of messages influence public perceptions of solar geoengineering. Figures 1 and 2 present the marginal means (MMs) of trust in the messenger and support for SG, respectively. Figures A.1 and A.2 in the Supplementary Information (SI) present the average marginal component effects (AMCEs) of these attributes. The AMCEs report the estimated effect of each attribute level relative to a baseline category, keeping all other factors constant. Unless otherwise noted, the effects reported below are statistically significant at the 95% confidence level, based on AMCEs estimates.

### Partisanship

Comparing copartisan sources (i.e., a messenger sharing the respondent’s political affiliation) to cross-partisan sources, we find that partisan identity strongly influences both trust in the messenger and support for SG. Figure 1 shows that copartisan sources increase Republicans’ trust in the messenger by about 1.03 points (from 3.04 to 4.07 on a scale of 0–10), approximately a 34% increase compared to trust levels with cross-partisan sources. Figure 2 indicates that receiving information from a copartisan source increases support for SG by about 0.37 points on a scale of 0–10 (from 3.48 to 3.85) among Republicans, roughly a 10% increase relative to a scenario in which information is provided by a cross-partisan source.

For Democrats, the pattern is similar. Receiving information from a copartisan source increases trust in the messenger by approximately 1.26 points (from 4.51 to 5.77 on a scale of 0–10), corresponding to approximately a 28% increase relative to cross-partisan sources. Copartisan sources increase support for SG by approximately 0.41 points on a scale of 0–10 (from 5.54 to 5.95), equivalent to approximately a 7% increase relative to cross-partisan sources.

### Occupational role

The occupational role of the messenger (researcher versus podcaster) is less influential. Among Republicans, there are no meaningful differences in trust or support based on the source’s occupation. For Democrats, trust in researchers is slightly higher than trust in podcasters (5.28 vs 5.00 on a scale of 0–10, or approximately 5.6% higher), but this difference does not extend to support for SG. These results suggest that the effects of occupation are modest and pale compared to the influence of partisanship.

### Framing

Message framing—whether SG is presented as complementary to existing efforts, as a substitute for emission reductions, or as a potential moral hazard—overall exerts limited influence. Among Republicans, framing does not meaningfully alter trust or support. For Democrats, the substitution frame slightly reduces trust in the messenger compared to the complementary and moral hazard frames by 7% (from 5.22 to 4.83) and 10% (from 5.34 to 4.83), respectively, while support remains relatively unaffected.

### Policy preferences

**Fig. 3:**
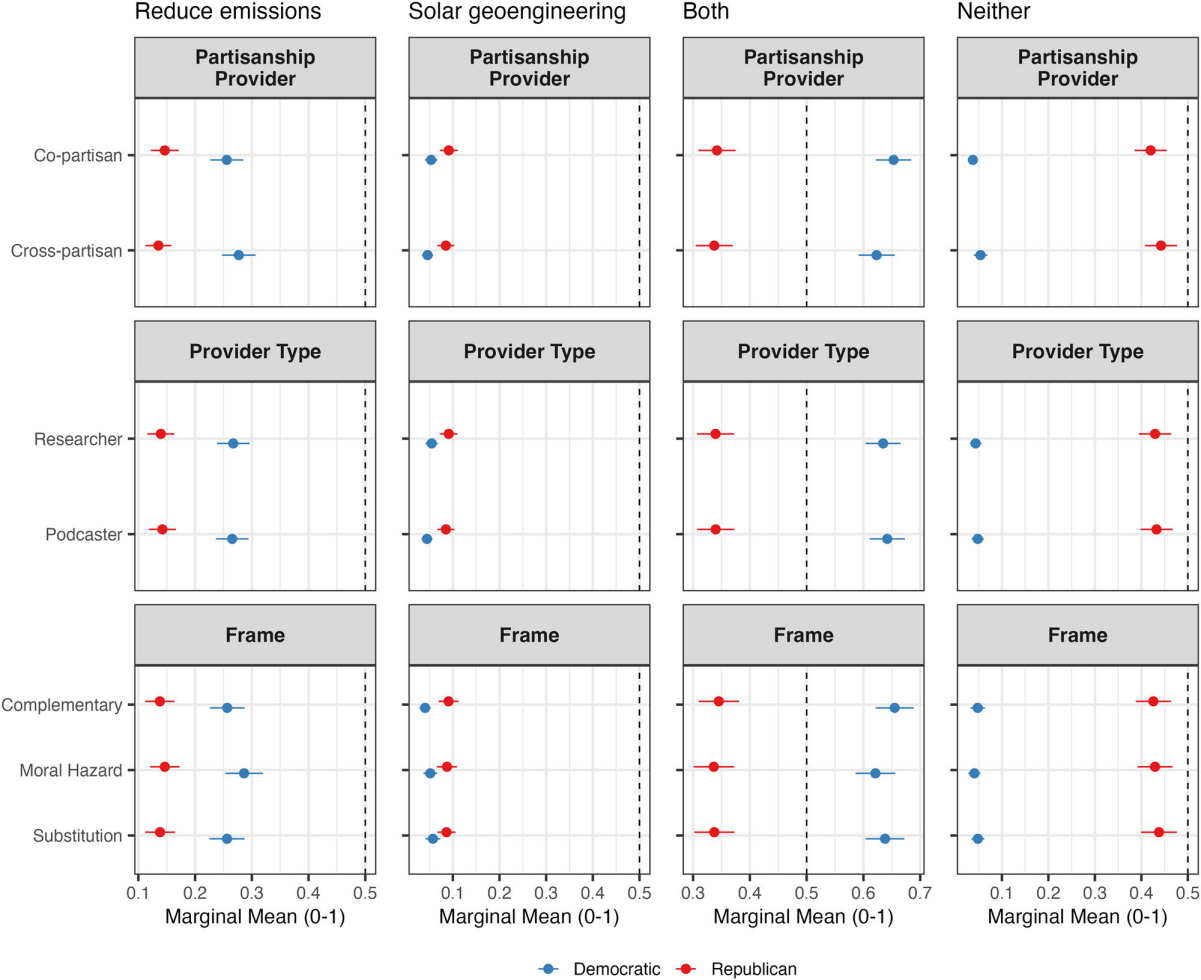
Marginal means of support for climate mitigation policies by respondent’s partisanship. Marginal means show the average outcome for each specific level of a conjoint attribute, averaging across all other attributes. Error bars represent 95% confidence intervals.

Policy preferences exhibit greater stability, reflecting ideological baselines that are less responsive to partisan cues or message framing. Figure 3 shows the marginal means for respondents’ preferred policy options, while Figure A.3 in the SI provides the AMCEs for these preferences.

As evident in both Fig. 3 and Fig. A.3, Republicans’ policy preferences remain largely unchanged across treatments. Their most common preference is to pursue neither SG nor emissions reductions, followed by supporting both, emissions reductions only, and SG alone. These preferences align closely with preexisting skepticism toward climate change mitigation efforts.

Democrats’ policy preferences are somewhat more responsive. Copartisan sources increase their likelihood of supporting “both” SG and emissions reductions by about 5% (from 0.62 to 0.65), while slightly decreasing support for “neither” (from 0.05 to 0.04, a 20% decrease). Relative to the complementary frame (the reference group), the moral hazard frame nudges Democrats slightly toward emissions reduction alone (an 8% increase from 0.25 to 0.27) and away from doing both (a 5% decrease from 0.65 to 0.62). The substitution frame modestly increases support for the pursuit of SG alone by 30% (from 0.05 to 0.07).

### Termination Shock

**Fig. 4:**
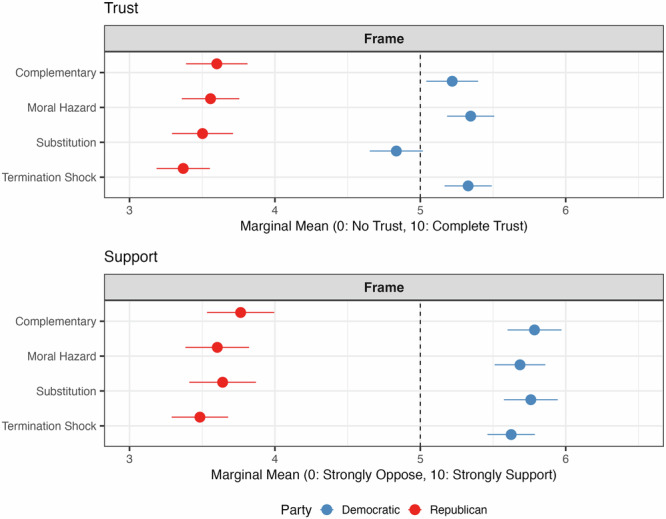
Marginal means of trust in the information source and support for SG, including the termination shock frame, by respondent’s partisanship. Marginal means show the average outcome for each specific level of a conjoint attribute, averaging across all other attributes. Error bars represent 95% confidence intervals.

**Fig. 5:**
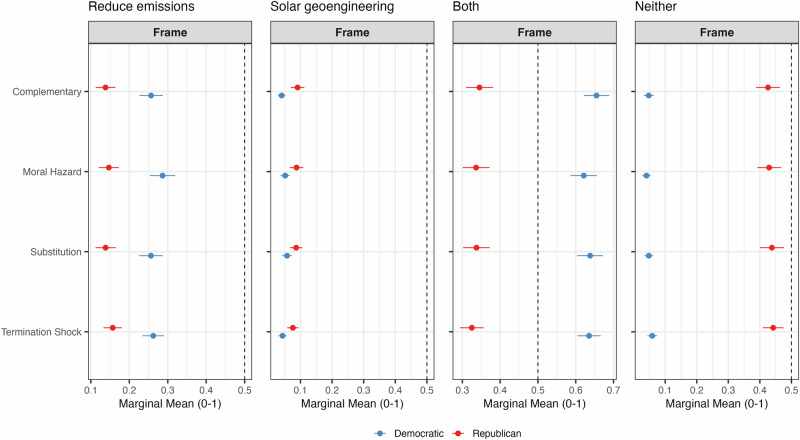
Marginal means of support for climate mitigation policies, including the termination shock frame, by respondent’s partisanship. Marginal means show the average outcome for each specific level of a conjoint attribute, averaging across all other attributes. Error bars represent 95% confidence intervals.

Figures 4 and 5 illustrate the effects of exposing respondents to the concept of termination shock—the rapid warming that could occur if SG were suddenly stopped. Unlike the earlier randomized frames (complementary, substitution, and moral hazard), the termination shock frame was introduced later as a sixth distinct scenario to evaluate its potential to change perceptions after exposure to previous frames.

Figure 4 presents the MMs for trust in the information source and support for SG, while Fig. A.4 in the SI provides the corresponding AMCEs. For Republicans, trust in the messenger is significantly lower under the termination shock frame compared to the moral hazard and complementary frames, with reductions of 5.3% (from 3.56 to 3.37) and 6.4% (from 3.60 to 3.37), respectively. Support for SG is also significantly lower under the termination shock frame compared to the substitution and complementary frames, with reductions of 4.3% (from 3.64 to 3.48) and 7.5% (from 3.76 to 3.48), respectively. The decrease in trust and support among Republicans in the termination shock condition may reflect a higher sensitivity to risks or discomfort with messaging that frames SG as less self-sufficient and more dependent on complementary mitigation efforts.

For Democrats, trust in the messenger under the termination shock frame is similar to trust under the moral hazard and complementary frames, and 10.3% higher compared to the substitution frame (from 4.83 to 5.33). The support for SG is slightly lower under the termination shock frame compared to the complementary frame, with a reduction of 2.9% (from 5.79 to 5.62), and it is similar to the support under the substitution and moral hazard frames.

As shown in Fig. 5 and A.5, policy preferences remain largely stable for both groups under the termination shock condition. These findings indicate that while the termination shock scenario can modestly alter trust and support—particularly for Republicans—it does not uniquely reshape overall policy preferences.

## Discussion

In this study, we investigate how different message frames of solar geoengineering—whether presented as a complement to existing climate mitigation efforts, as a substitute, or as a potential moral hazard—and the identity of the information sender (researcher or podcaster, Republican or Democrat) influence public attitudes toward SG. Using a high-quality large-n representative survey from YouGov, our study addresses limitations in prior research, offering robust and generalizable insights.

Our results reveal that the partisan alignment between the information source and the respondent is a dominant factor in shaping trust and support for SG. Specifically, Republicans exhibit 10% higher support for SG and 34% greater trust in the communicator when information is provided by copartisan sources compared to cross-partisan sources. Similarly, Democrats show 7% higher support for SG and a 28% increase in trust in the messenger when the information comes from a source aligned with their political affiliation.

The occupation of the messenger—whether they are researchers or podcasters—has limited influence on respondents’ opinions. Among Democrats, trust in researchers is slightly higher than trust in podcasters, but this difference does not extend to support for SG. For Republicans, the occupation of the messenger does not have a significant effect. This suggests that perceptions of credibility are primarily shaped by partisan alignment rather than professional expertise.

Message framing, whether SG is framed as a complement, substitute, or moral hazard, has a similar limited impact. Although Democrats exhibit a slight reduction in trust under the substitution frame compared to the complementary and moral hazard frames, support for SG remains stable across frames. Among Republicans, framing has no meaningful effect on trust or support. These results highlight the dominance of partisan identity in filtering and interpreting information about SG, even in the presence of varied frames.

Despite the influence of partisan alignment on trust in the messenger and support for SG, we find that policy preferences generally revert to ideological baselines. For Republicans, the modal response remains consistent with their ideological predispositions, a preference for investing neither in SG nor in emissions reductions. In contrast, while Democrats exhibit a more nuanced response, their policy preferences also largely reflect their ideological beliefs, with a preference for pursuing both SG and emission reductions.

The introduction of the termination shock frame, a scenario emphasizing the potential consequences of abruptly halting SG while continuing CO_2_ emissions, leads to a slight shift in Republicans’ trust and support for SG. However, the policy preferences for both Republicans and Democrats remain largely stable.

Overall, our results reveal that the partisan alignment between the source of information and the respondent plays a critical role in shaping public perceptions of solar geoengineering. In a politically polarized environment, partisan cues outweigh other factors, such as the messenger’s professional background or the content of the message. These findings highlight the difficulty of depolarizing the discourse on emerging climate technologies, where deeply entrenched partisan identities filter how information is received and interpreted.

The limited impact of the framing observed in this study also raises important questions for climate communication strategies. While framing has been shown to influence attitudes in some contexts, its effectiveness appears constrained in highly polarized domains like US climate politics. This suggests that communication strategies must account for the primacy of partisan cues and the broader ideological context in which messages are delivered. Efforts to engage the public in SG may need to prioritize building trust across partisan lines to reduce the dampening effects of polarization on framing.

Using a conjoint experimental design, our study captures the complexity of real-world communication environments. Climate-related information rarely arrives in isolation; instead, audiences encounter simultaneous cues about the source’s partisan identity, the technological solution proposed, and the broader policy narrative. Our findings demonstrate how partisan identity can overshadow framing effects—a pattern that might remain hidden if either factor were studied in isolation. In particular, this aligns with other recent work showing that even studies focusing exclusively on framing often find limited effects on public engagement with climate solutions such as SG^43^.

At the same time, we do not claim that framing is universally inconsequential. Our findings pertain to a specific, highly polarized domain (US climate politics) and a relatively unfamiliar technology (SG), where entrenched partisan identities may quickly preempt subtle framing effects. These results highlight the conditional nature of framing: in this setting, partisan cues dominate, but under different conditions, such as lower polarization, stronger frames, or alternative informational environments, framing may play a more significant role.

More broadly, as with any survey experiment, respondents here were exposed to a controlled set of messages and messengers that cannot fully replicate real-world information environments. Thus, one should use caution when generalizing these results to broader media ecosystems where individuals might encounter multiple conflicting cues, policy details, and spontaneous elite rhetoric. Moreover, although our large, nationally representative sample bolsters internal validity, we cannot definitively test whether the observed patterns would persist under more prolonged exposures or in different contexts. Finally, our focus on a specific set of frames (complement, substitute, moral hazard) and partisan identities (Democratic vs. Republican) omits other potentially influential factors that could emerge in practice.

In sum, our goal was to highlight how, in a highly polarized political context, partisan cues can overshadow message framing for an emerging climate technology that has yet to enter mainstream discourse. We hope these findings serve as a starting point for deeper investigations into public engagement with solar geoengineering, including longitudinal studies, cross-national comparisons, and mixed-methods research that could further examine how and why polarization emerges around novel climate interventions.

## Methods

We employ a conjoint experimental design to evaluate how different frames of solar geoengineering (complement, substitution, moral hazard) and distinct messenger attributes (political affiliation and occupation) jointly influence public attitudes toward SG. Conjoint analysis is increasingly prevalent in social science research due to its ability to approximate decision-making processes in which individuals encounter multiple, simultaneously varying attributes^57–59^.

Conjoint experiments offer several methodological advantages. First, rather than focusing on a single attribute at a time, conjoint designs systematically vary multiple features. In real-world scenarios, individuals are rarely exposed to isolated attributes, such as a policy frame or a messenger’s partisanship, in a vacuum. Instead, they encounter a combination of cues embedded within a broader media and political context. By systematically presenting these combinations, conjoint experiments provide a more comprehensive approximation of the informational complexity people face in everyday political reasoning. In addition, conjoint designs help identify which factors are most influential and how these factors interact.

To test our predictions, we conducted a survey with 2,123 US registered voters, recruited through the survey firm YouGov in December 2023. Table A.1 in the SI provides an overview of the sample demographics, which align closely with population benchmarks. Before data analysis, we preregistered our study protocol on the Open Science Framework (OSF) at 10.17605/OSF.IO/GR8BK. Participants were selected from YouGov’s opt-in panel to be representative of the US voter population. YouGov employs a stratified random sampling method, ensuring that the sample reflects demographic factors such as age, race/ethnicity, gender, education, geographic region, and 2020 presidential vote. The population targets for these strata were derived from model estimates based on the 2019 American Community Survey, the November 2020 Current Population Survey, and the TargetSmart Voter Files. This approach produced a representative cross-section of respondents, enhancing the generalizability of our findings. Furthermore, data collection and analysis procedures were reviewed and deemed exempt from the Institute Research Board at the California Institute of Technology (IR22-1220).

### Analytical strategy

The conjoint experiment serves as our primary empirical component and is divided into two parts. We use “trust”, “support for SG”, and “policy preference” as our dependent variables, which we regress on three attributes: whether the source of information is copartisan or cross-partisan, whether they are researchers or podcasters, and the framing (complement, moral hazard, or substitute). “Trust” and “support for SG” are continuous variables ranging from 0 to 10, capturing how much the respondent trusts the information source and their level of support for solar geoengineering. The “policy preference” variable has four categories, measuring whether the respondent believes the US should invest in reducing emissions only, pursuing SG only, doing both, or doing neither. We treat each category as a dummy variable and run four separate regression models. To account for partisan effects, we interact the three attributes with the partisanship of the respondents, focusing on Democrats and Republicans. To increase statistical power, we classify Independents leaning toward the Democratic Party as Democrats and Independents leaning toward the Republican Party as Republicans. This approach results in a sample of 1828 respondents. Results remain consistent when Independents are excluded from partisan groupings.

Each regression model includes 9140 observations—calculated as 1828 respondents across five rounds of the conjoint experiment. We present marginal means (MM) in our primary results to align with our research objectives. Although causal average marginal component effects (AMCEs) are useful for isolating the relative impact of specific attributes, measuring how individual factors affect trust, support for SG, or policy preferences compared to a baseline, MMs offer a more comprehensive perspective. By representing overall levels of trust, support, or preferences for each attribute without depending on a reference category, MMs allow us to better capture absolute attitudes in different scenarios, such as variations in the frame or the attributes of the messenger^59^.

This approach is particularly advantageous when analyzing differences between subgroups. MMs are not influenced by changes in the baseline category, making them an effective tool to compare absolute levels of trust and support between partisan groups^59^. While the main analyses focus on MMs for their interpretability and descriptive value, AMCEs, which represent causal estimates, are included in the SI. To account for the repeated measures design of the conjoint experiment, standard errors are clustered by respondent^59^.

In the second part of the experiment, we introduce the “termination shock” frame. Here, we use conjoint analysis and the same outcome variables, but include only one attribute, the frame (complement, moral hazard, substitute, or termination shock), interacted with the respondent’s partisanship. Each regression model in this part includes 10,968 observations, calculated as 1828 respondents across five rounds of the conjoint experiment plus the final post-conjoint frame. All analyses were performed using R, specifically the *cregg* package.

Although conjoint experiments cannot perfectly replicate real-world information environments, they provide a structured way to assess how multiple attributes jointly influence attitudes^58,59^. However, concerns may arise about order effects, primacy effects, demand effects, and the realism of repeated exposures. To address these issues, we performed additional robustness checks. First, we analyzed only the first vignette shown to respondents—before any potential learning or carryover effects could emerge—and found that our key patterns remain intact. Second, we examined subgroups with weaker partisan attachments (e.g., true Independents), and again, framing effects remained minimal, indicating that these findings are not solely due to participants simplifying their evaluations through partisan heuristics. Finally, research on demand effects in survey experiments has shown that such biases are often small^60^. Thus, while no design can fully eliminate potential artifacts, these additional analyses (detailed in the SI) bolster our confidence that the core findings are robust and not driven solely by design choices or respondent “satisficing”.

Finally, while we did not include explicit manipulation checks for this experiment, we took steps to ensure that our findings were not driven by differences in attentiveness. YouGov employs rigorous quality controls and our sample comes from a professional survey provider with a strong track record of reliable data quality. To rule out that our findings are driven by inattentive respondents, in the SI, we analyzed response times to differentiate between inattentive (below the 25th percentile) and attentive (above the 75th percentile) respondents. This robustness check revealed no significant differences in the effects of partisanship, provider type, or message framing at all levels of attention.

### Research design

#### Socio-demographic section

Participants first answered sociodemographic questions and were asked about their awareness of solar geoengineering, after being provided with a brief definition of the technology.

Solar geoengineering (SG) is an emerging technology proposed to cool the Earth’s atmosphere by increasing the amount of sunlight reflected from Earth or by reducing the trapping of outgoing thermal radiation.

How much have you heard about solar geoengineering in the last year?

- A lot
- Some
- A little
- Nothing

We deliberately kept this initial framing brief to isolate the effects of partisan identity and message framing in subsequent treatments. This approach minimizes the potential for confounding effects that could arise from extensive contextual details or pre-treatment priming. Although this choice improves parsimony and reduces cognitive load on respondents, we acknowledge that it also limits the ecological validity of the study. Public attitudes toward SG in real-world settings can evolve differently when exposed to richer media discourse, in-depth reporting, or policy debates. Future research could explore how the inclusion of more detailed background information affects the engagement of respondents with SG.

#### Main conjoint experiment

The respondents were then shown five repetitions of the treatment, each consisting of two key components.

**Information senders:** There are four hypothetical senders, each with a distinct political alignment and role:

- Jane Smith is a researcher from Brookings, a liberal think tank that is generally aligned with the Democratic party;
- Michelle Davis is a researcher from the American Enterprise Institute, a conservative think tank that is generally aligned with the Republican party;
- Sarah Anderson hosts the Sarah Anderson show, a prominent conservative podcast that is generally aligned with the Republican party;
- Julia Wilson hosts the Julia Wilson show, a prominent liberal podcast that is generally aligned with the Democratic party.

**Framing of solar geoengineering:** Each sender presented solar geoengineering in one of three frames:

- Substitution frame: this emerging technology offers a solution that can effectively cool the Earth’s atmosphere without requiring everyone to continue reducing emissions and drastically change their lifestyles.
- Complementary frame: this emerging technology offers a solution that can effectively cool the Earth’s atmosphere, helping reduce some of the risks of climate change. It complements our ongoing efforts of emission reduction, carbon removal and sequestration, and adaptation strategies.
- Moral Hazard Frame: while solar geoengineering offers hope for a technological fix to cool the Earth’s atmosphere, relying on this solution may delay or even halt urgently needed efforts to reduce emissions.

Here is an example of one of the five conjoint scenarios that a respondent may see:

Jane Smith is a researcher from Brookings, a liberal think tank that is generally aligned with the Democratic party. In her most recent publication, she discussed solar geoengineering. She argued that this emerging technology offers a solution that can effectively cool the Earth’s atmosphere, helping reduce some of the risks of climate change. It complements our ongoing efforts of emission reduction, carbon removal and sequestration, and adaptation strategies.

All senders were presented with female-sounding names to maintain the perceived gender of the source constant in all treatments. This decision reduces the potential for gender cues to influence trust or support for the source, ensuring that observed differences are driven by political alignment and framing. However, this design choice means that we cannot assess whether the effects might differ if the source were male. Future research could vary the source’s gender to explore potential interactions between perceived gender and source credibility.

#### Post-conjoint questions

After each conjoint scenario, respondents answered the following questions:

1. How much do you trust this information source? (0–10), where 0 = “Do not trust at all” and 10 = “Trust completely.”
2. How likely are you to support solar geoengineering? (0–10), where 0 = “Not at all likely” and 10 = “Very likely.”
3. Do you think that the US should invest mainly in reducing carbon emissions, invest mainly in solar geoengineering techniques, both, or neither?

The 0–10 scales were fully labeled at key points. Specifically, the endpoints of each scale were anchored to meaningful verbal descriptors (e.g., “Do not trust at all” and “Trust completely” for trust; “Not at all likely” and “Very likely” for SG support).

#### Final prompt on termination shock

After completing the main conjoint scenarios, participants received a final vignette discussing the risks of “termination shock”—the abrupt warming that could occur if SG efforts were suddenly stopped while carbon dioxide levels remained high. An example vignette reads:

Michelle Davis, a researcher from the American Enterprise Institute, a conservative think tank that is generally aligned with the Republican party, also added that: If we pursue solar geoengineering (SG), we must continue reducing emissions and removing and sequestering carbon from the atmosphere. If we kept constant or increased our emissions while pursuing SG, one of the biggest risks would be suddenly stopping SG because stopping it would cause any carbon dioxide in the air to rewarm the planet faster than before, leading to catastrophic consequences.

#### Post-final prompt questions

Participants rated their trust in the information source and their likelihood of supporting SG on a scale from 0 to 10. They also indicated their preference for investing in reducing carbon emissions, solar geoengineering techniques, both, or neither.

## Supplementary information

Appendix.

## Data Availability

Upon publication, the code and data necessary to reproduce the results reported in this paper will be made available in a permanent and public data repository, subject to any limitations imposed by human subjects considerations.
