# Supplementary material for: Partisanship overcomes framing in shaping solar geoengineering perceptions: Evidence from a conjoint experiment
Source: NPJ Clim Action. 2025 Mar 24;4(1):29. doi: 10.1038/s44168-025-00236-3 (PMC11932920; doi:10.1038/s44168-025-00236-3)

A Online Supplementary Information

Survey and Pre-Analysis Plan (PAP)

This study was pre-registered with OSF at https://doi.org/10.17605/OSF.IO/GR8BK on January 2, 2024, prior to analyzing any data from YouGov. The survey was conducted between December 13 and December 20, 2023, and included 2,123 respondents from the United States. The sample was recruited by YouGov and was representative based on region, age, education, gender, race, and voting behavior in the last election. The conjoint experiment was executed as pre-registered, where respondents were exposed to randomized scenarios of partisan alignment, occupational roles, and frames for solar geoengineering (SG).

In the PAP we originally proposed seven hypotheses (H1–H7) focusing on the effects of partisanship, occupational role, framing (complement, substitute, moral hazard), and termination shock on respondents' trust and support for solar geoengineering. In the final manuscript, for clarity and parsimony, we consolidated the original seven hypotheses into four. Our new H1 merges the logic of original H1 and H2, examining how Republicans and Democrats respond to copartisan versus cross-partisan sources. Our new H2 draws on original H1 and H2's focus on the occupational identity of the source (researcher vs. podcaster). Our new H3 on framing effects synthesizes original H3, H4, and H5. Our new H4 (Termination Shock) covers original H6 and H7, both of which examined how learning about the abrupt-warming ("termination shock") scenario might alter support for SG and policy preferences, with one important deviation. As outlined in the PAP, a control group was initially included in the design to compare baseline support for SG with support after exposure to the termination shock frame. However, we identified a discrepancy in response options for two key questions in the control group. These questions included a "Don't know" option that was absent in the conjoint experiment. To maintain consistency in response formats and avoid potential bias in comparisons, we made a minor deviation from the PAP and excluded the control group from the final analysis. As a result, we assess termination shock effects solely within the conjoint framework, presenting the termination shock information as a final vignette. Throughout, we estimate and report marginal means and average marginal component effects from linear regressions on our conjoint data, consistent with standard practice for conjoint experiments.

Table A.1: Descriptive statistics of participant demographics

**Variable**	**Count (Percentage)**
Gender	
Female	1,150 (54%)
Male	973 (46%)
Age Group	
18 to 29	207 (9.8%)
30 to 44	407 (19%)
45 to 64	865 (41%)
65 or older	644 (30%)
Education	
HS or less	605 (28%)
Some college	683 (32%)
College grad	535 (25%)
Postgrad	300 (14%)
Region	
Northeast	382 (18%)
Midwest	503 (24%)
South	798 (38%)
West	440 (21%)
Party ID	
Democratic	779 (37%)
Republican	662 (31%)
Other	682 (32%)
Party ID (with leaners)	
Democratic	946 (44.5%)
Republican	882 (41.5%)
Other	295 (14%)

Figures

Figure A.1: Average marginal component effects (AMCEs) of trust in the information source by respondent's partisanship. AMCEs measure the causal effect of changing a specific level of a conjoint attribute on support, relative to the baseline level. Error bars represent 95% confidence intervals.

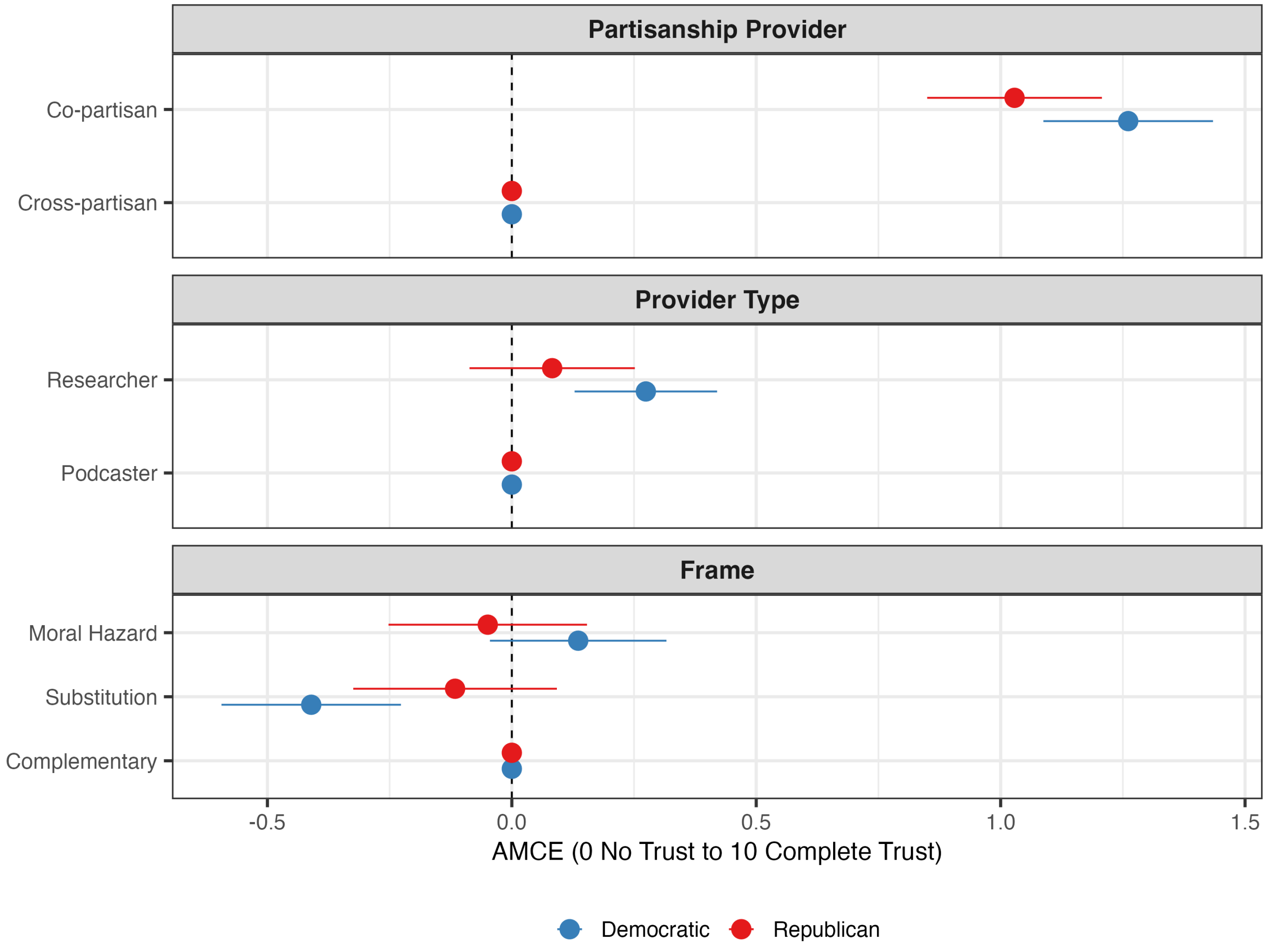

Figure A.2: Average marginal component effects (AMCEs) of support for SG by respondent's partisanship. AMCEs measure the causal effect of changing a specific level of a conjoint attribute on support, relative to the baseline level. Error bars represent 95% confidence intervals.

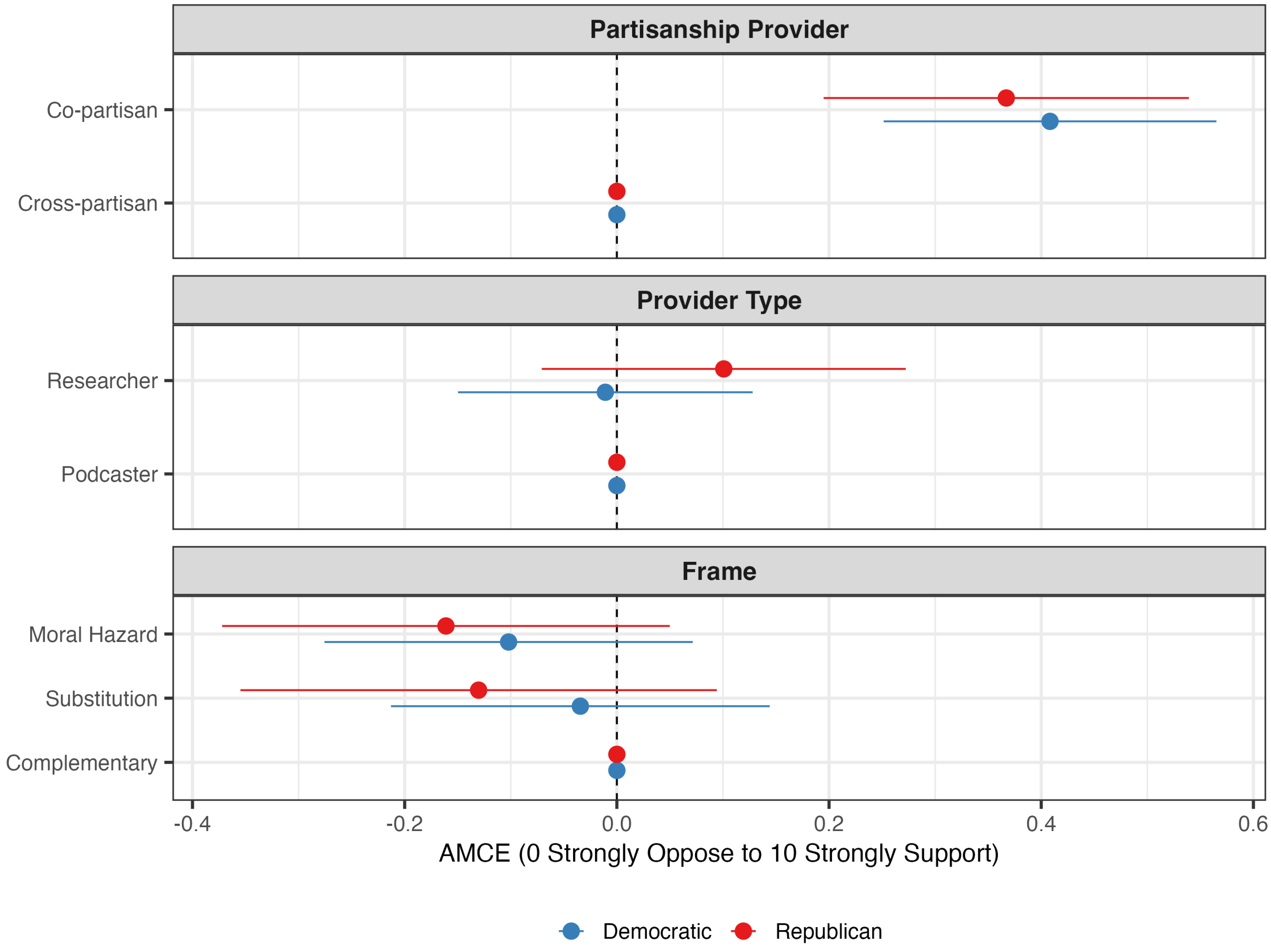

Figure A.3: Average marginal component effects (AMCEs) of policy preferences by respondent's partisanship. AMCEs measure the causal effect of changing a specific level of a conjoint attribute on support, relative to the baseline level. Error bars represent 95% confidence intervals.

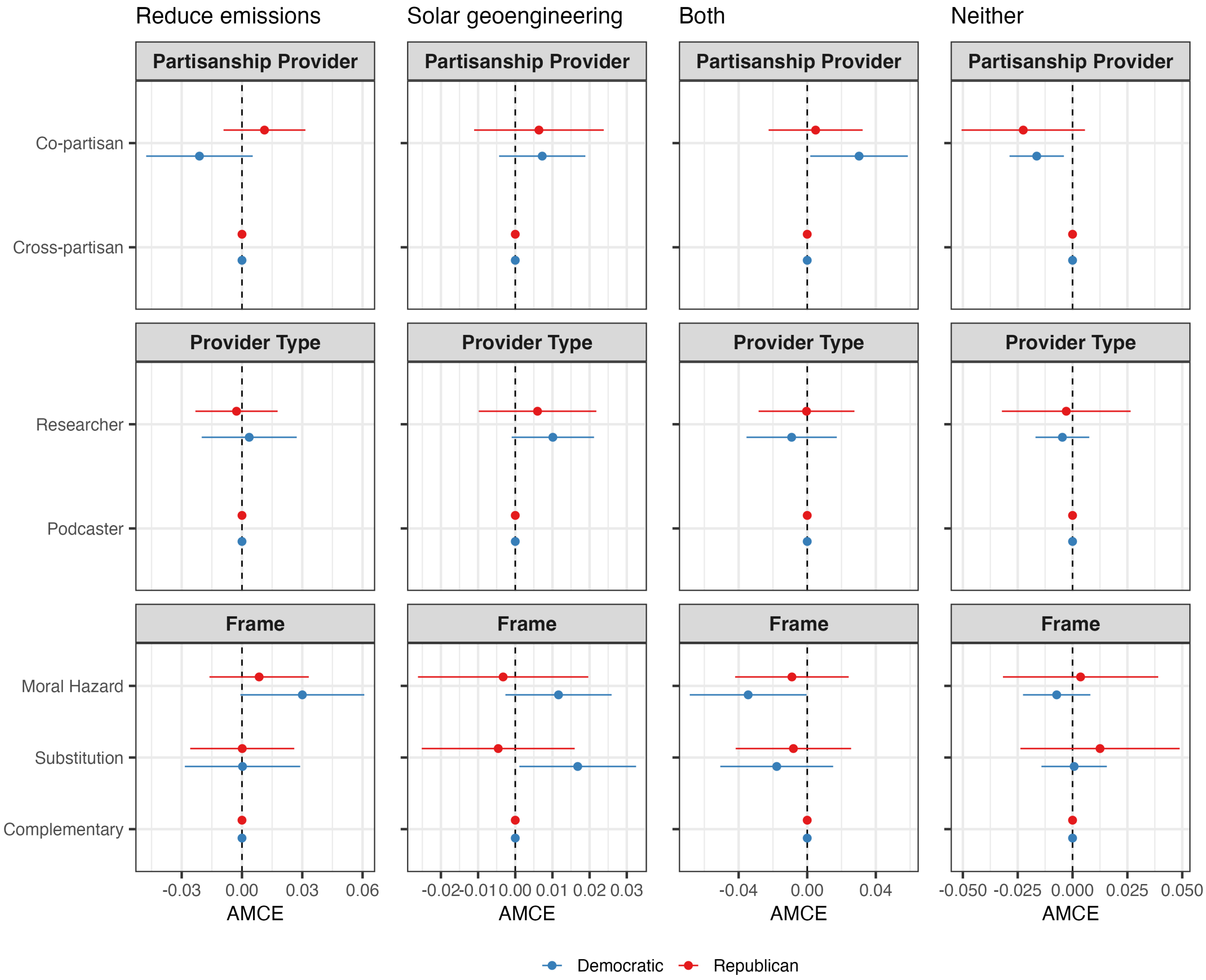

Figure A.4: Average marginal component effects (AMCEs) of trust in the information source and support for SG, including the termination shock frame, by respondent's partisanship. AMCEs measure the causal effect of changing a specific level of a conjoint attribute on support, relative to the baseline level. Error bars represent 95% confidence intervals.

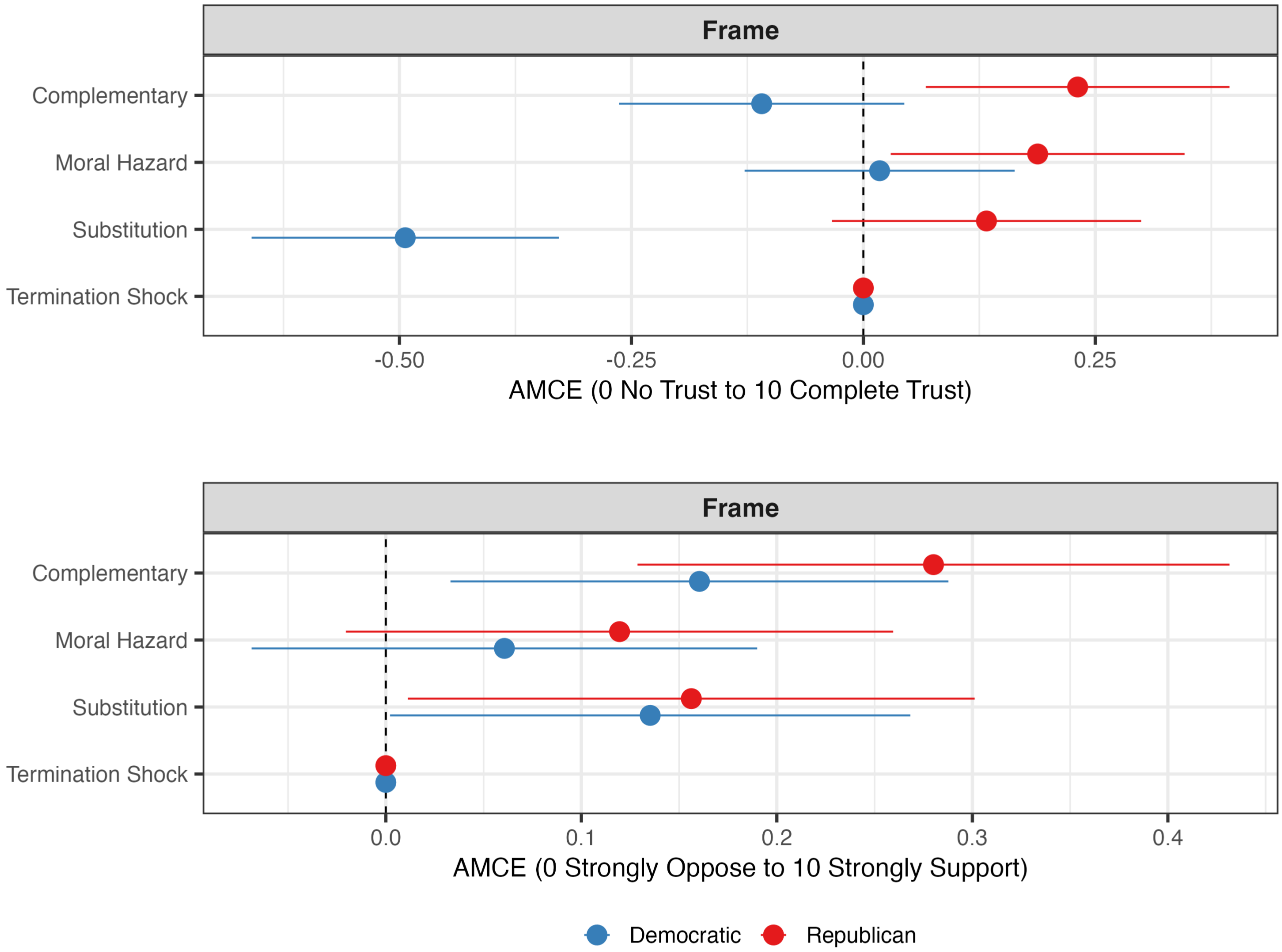

Figure A.5: Average marginal component effects (AMCEs) of policy preferences, including the termination shock frame, by respondent's partisanship. AMCEs measure the causal effect of changing a specific level of a conjoint attribute on support, relative to the baseline level. Error bars represent 95% confidence intervals.

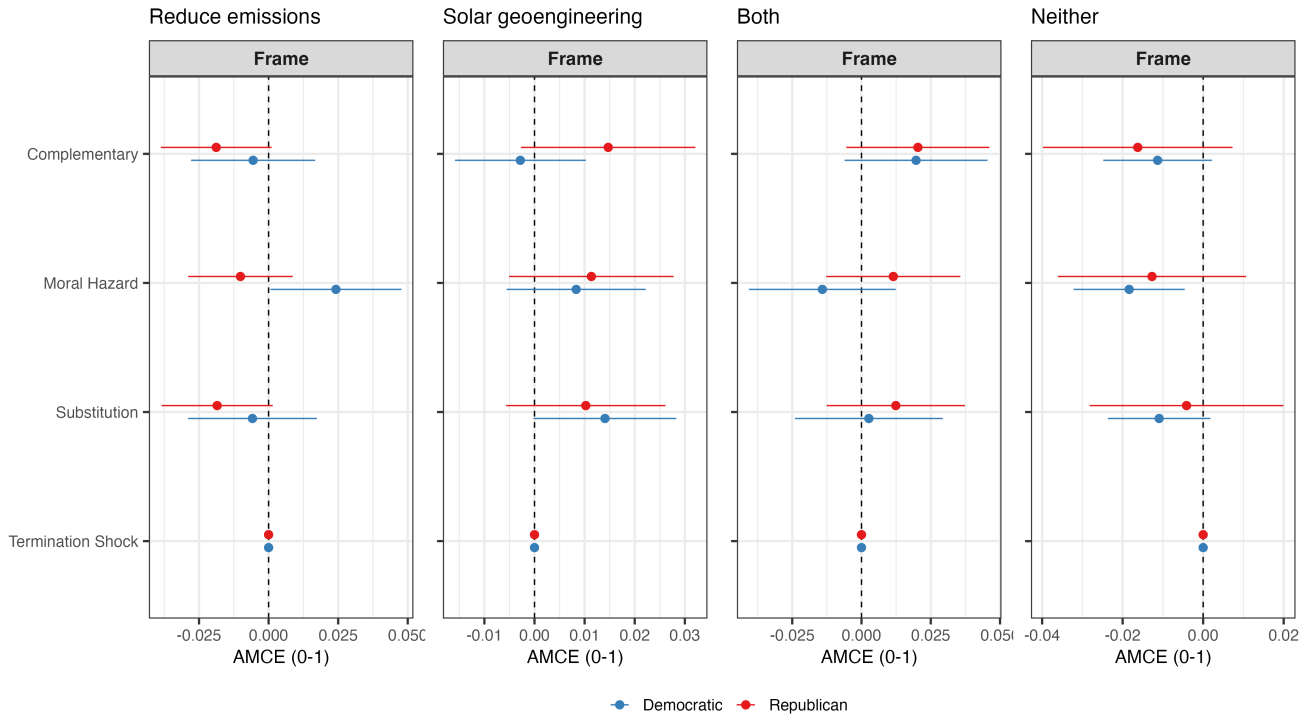

Robustness Checks

In this section, we present a series of robustness checks designed to ensure that our findings are not driven by potential order effects, respondent inattentiveness, or the particular combination of frames and sources included in the main analysis. First, we focus on the initial ("frame 1") exposure only, to assess whether the patterns we observe persist when respondents encounter each attribute level for the first time, thus minimizing concerns about learning, carryover, or consistency pressures. Second, we incorporate Independents more explicitly, examining whether the absence of clear partisan identification changes the relative influence of framing or source attributes. Finally, we distinguish between attentive and inattentive respondents, testing whether our core results hold even when restricting the sample to those most likely to have engaged deeply with the information presented. As the figures below illustrate, these additional analyses confirm that our key conclusions—particularly the dominance of partisan cues over framing and the limited impact of source occupation—are robust across these different specifications and subgroups.

Frame 1 only

Figure A.6: Predicted trust in the information source for frame 1 only by respondent's partisanship. Error bars represent 95% confidence intervals.

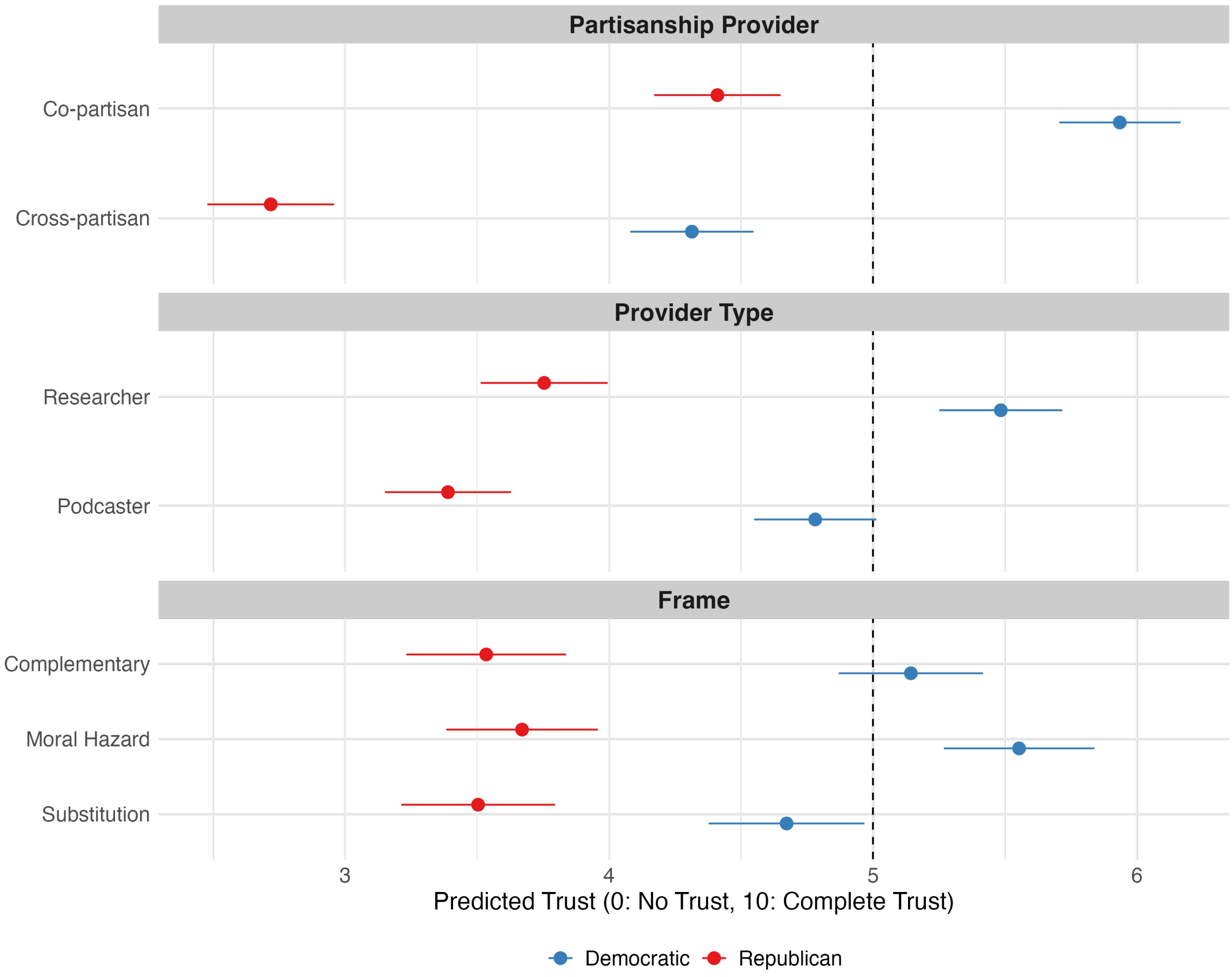

Figure A.7: Predicted support for SG for frame 1 only by respondent's partisanship. Error bars represent 95% confidence intervals.

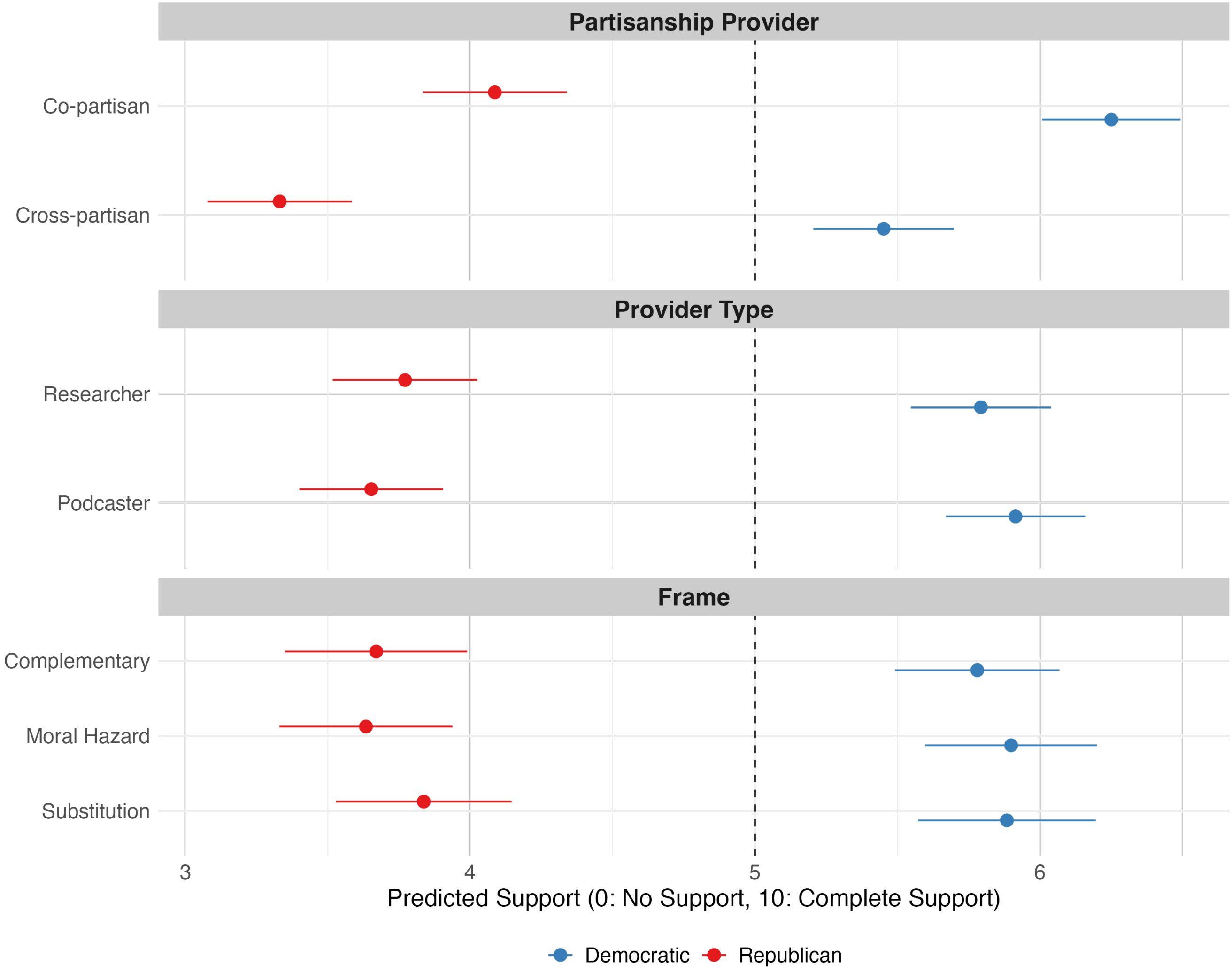

Figure A.8: Predicted support for climate mitigation policies for frame 1 only by respondent's partisanship. Error bars represent 95% confidence intervals.

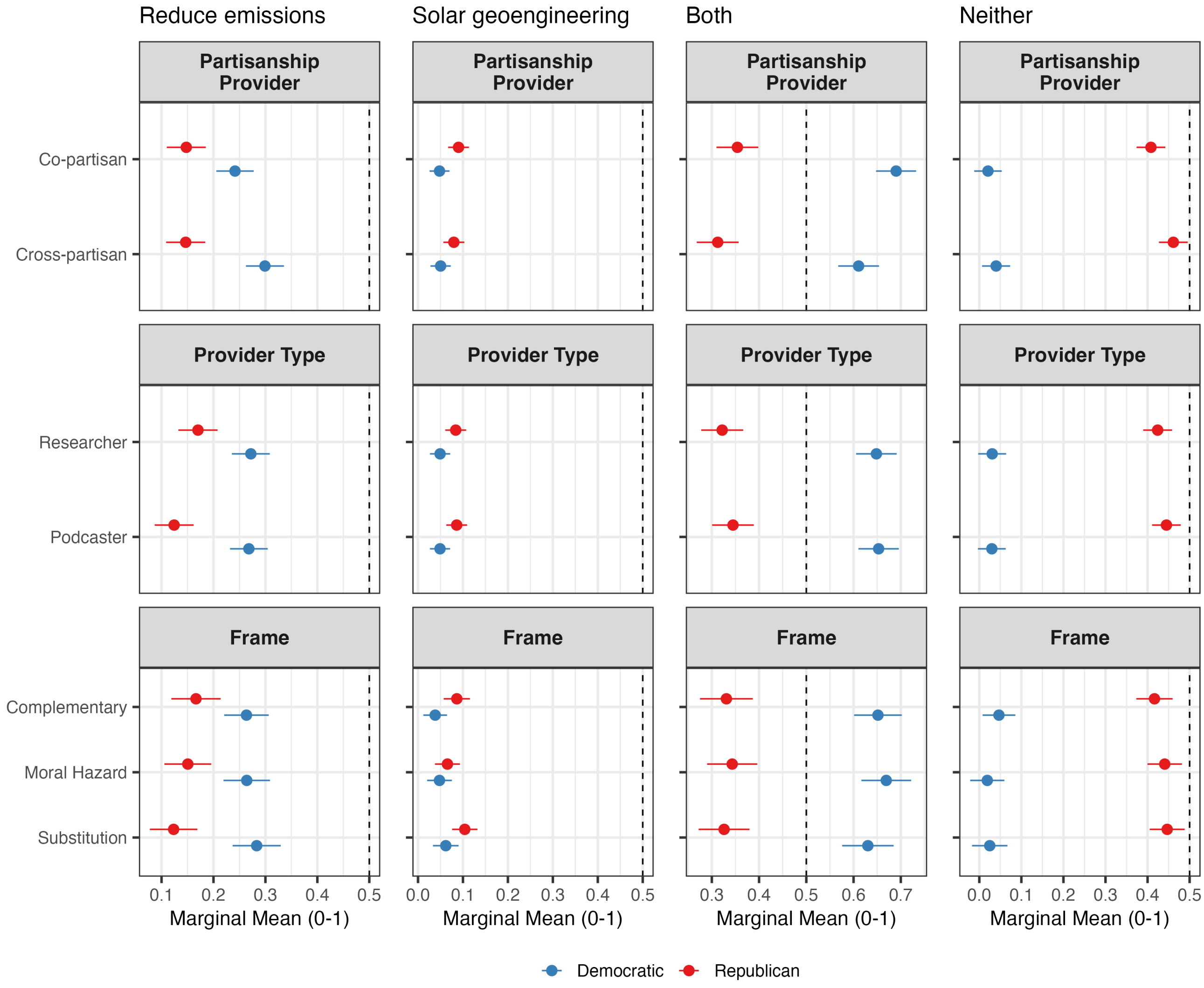

Independents

Figure A.9: Marginal means of trust in the information source, including Independents, by respondent's partisanship. Marginal means show the average outcome for each specific level of a conjoint attribute, averaging across all other attributes. Error bars represent 95% confidence intervals.

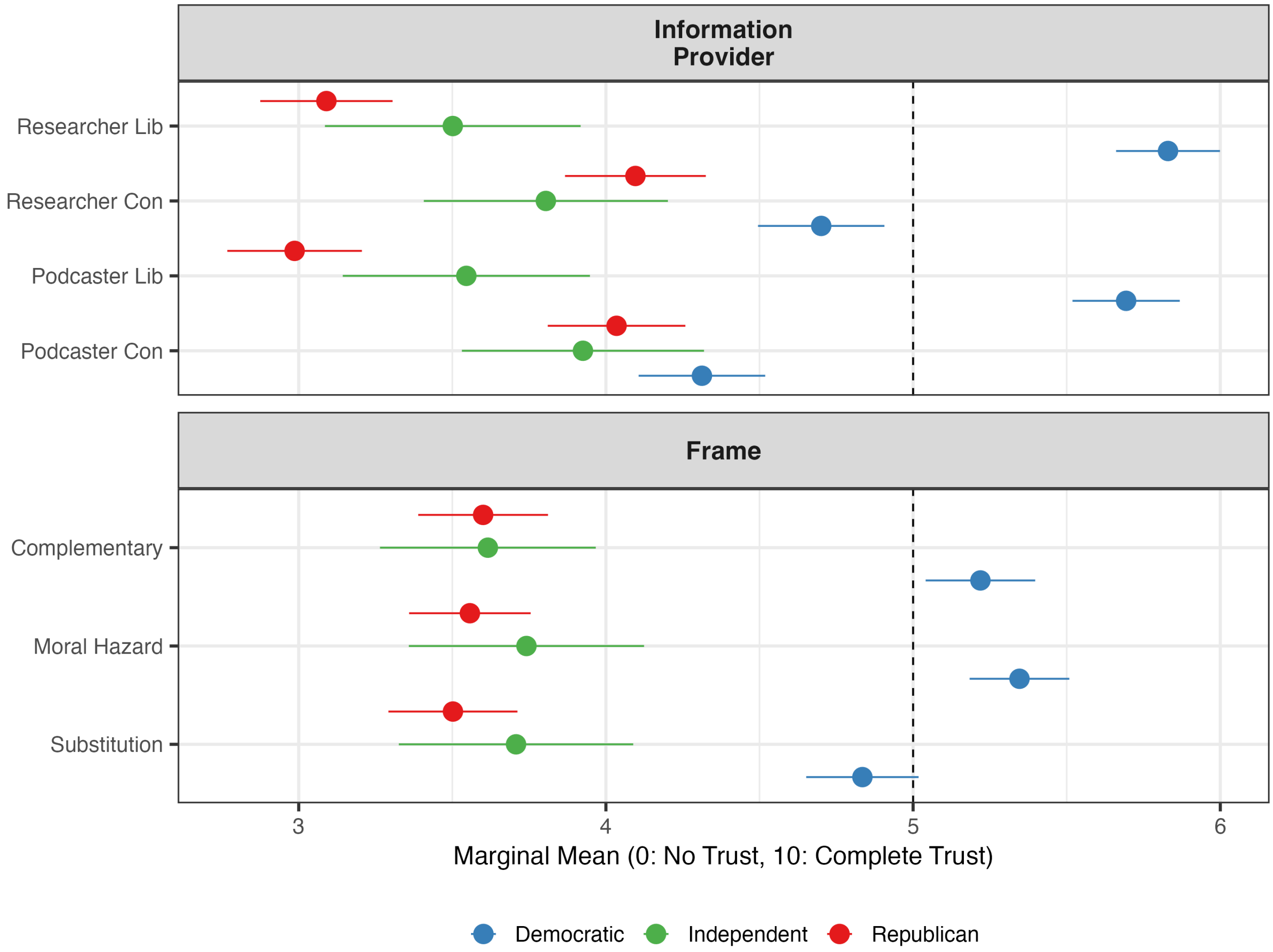

Figure A.10: Marginal means of support for SG, including Independents, by respondent's partisanship. Marginal means show the average outcome for each specific level of a conjoint attribute, averaging across all other attributes. Error bars represent 95% confidence intervals.

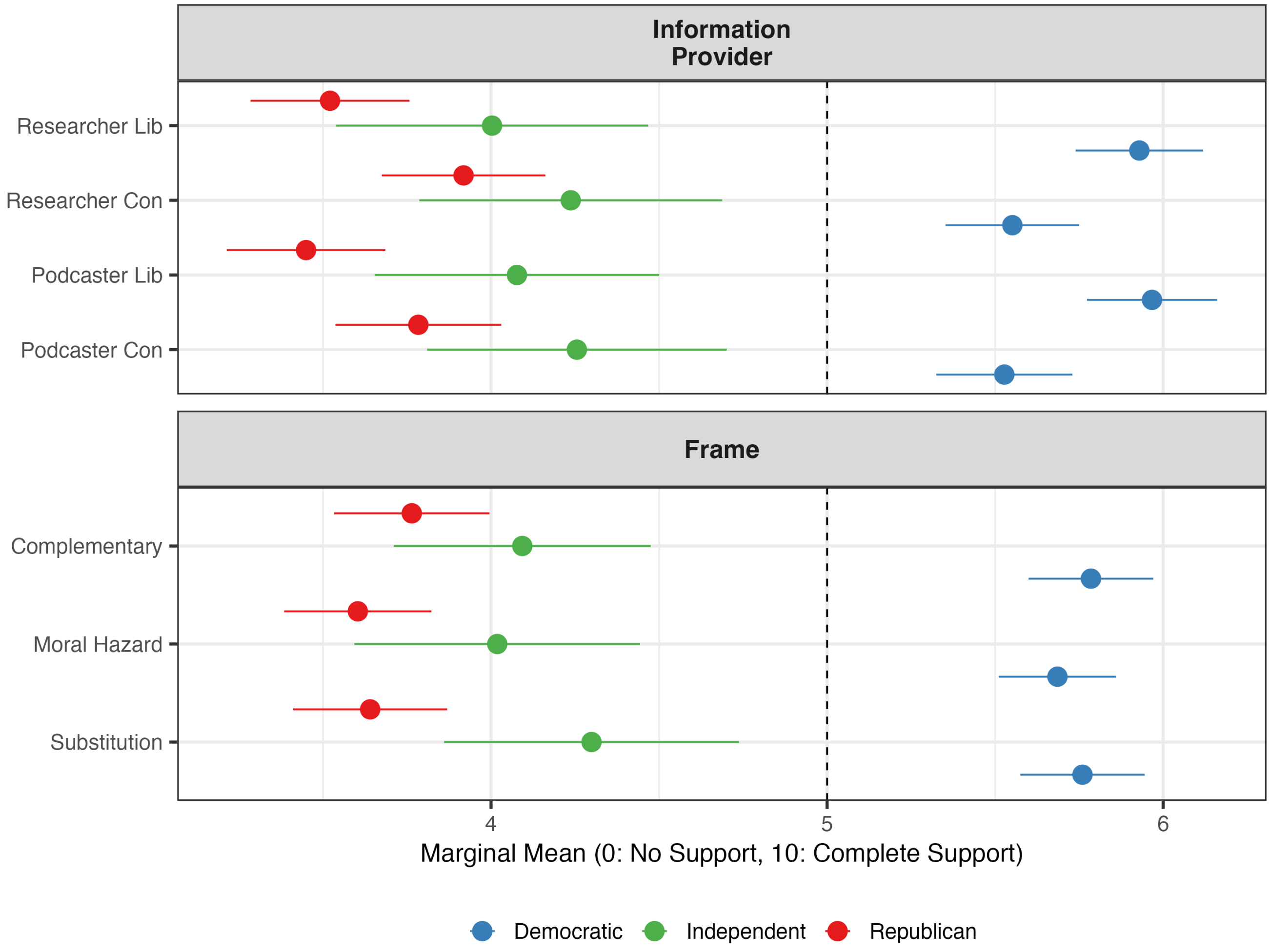

Figure A.11: Marginal means of support for climate mitigation policies, including Independents, by respondent's partisanship. Marginal means show the average outcome for each specific level of a conjoint attribute, averaging across all other attributes. Error bars represent 95% confidence intervals.

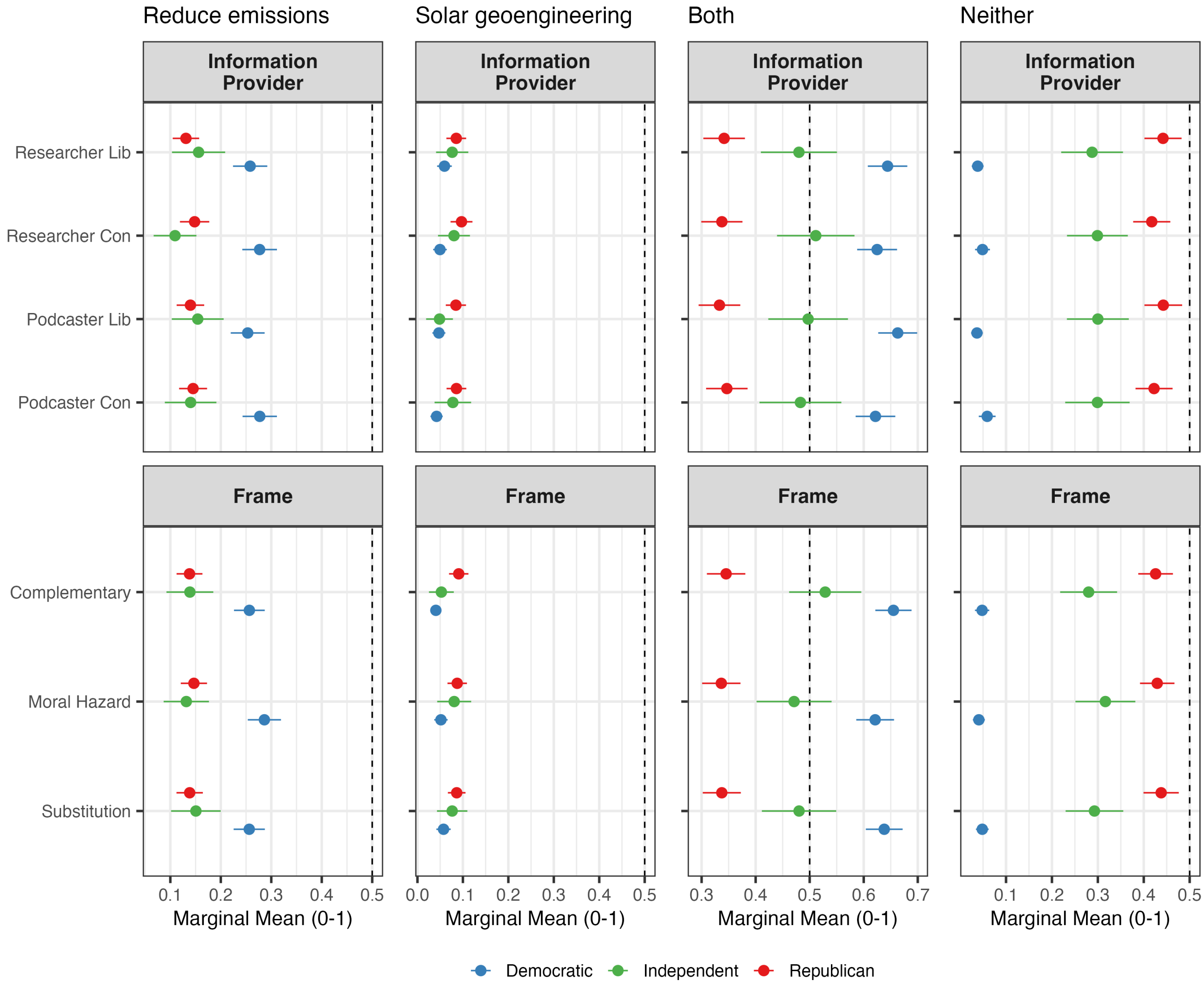

Attentiveness

Figure A.12: Marginal means of trust in the information source by attentiveness and partisanship. Marginal means show the average outcome for each specific level of a conjoint attribute, averaging across all other attributes. Error bars represent 95% confidence intervals.

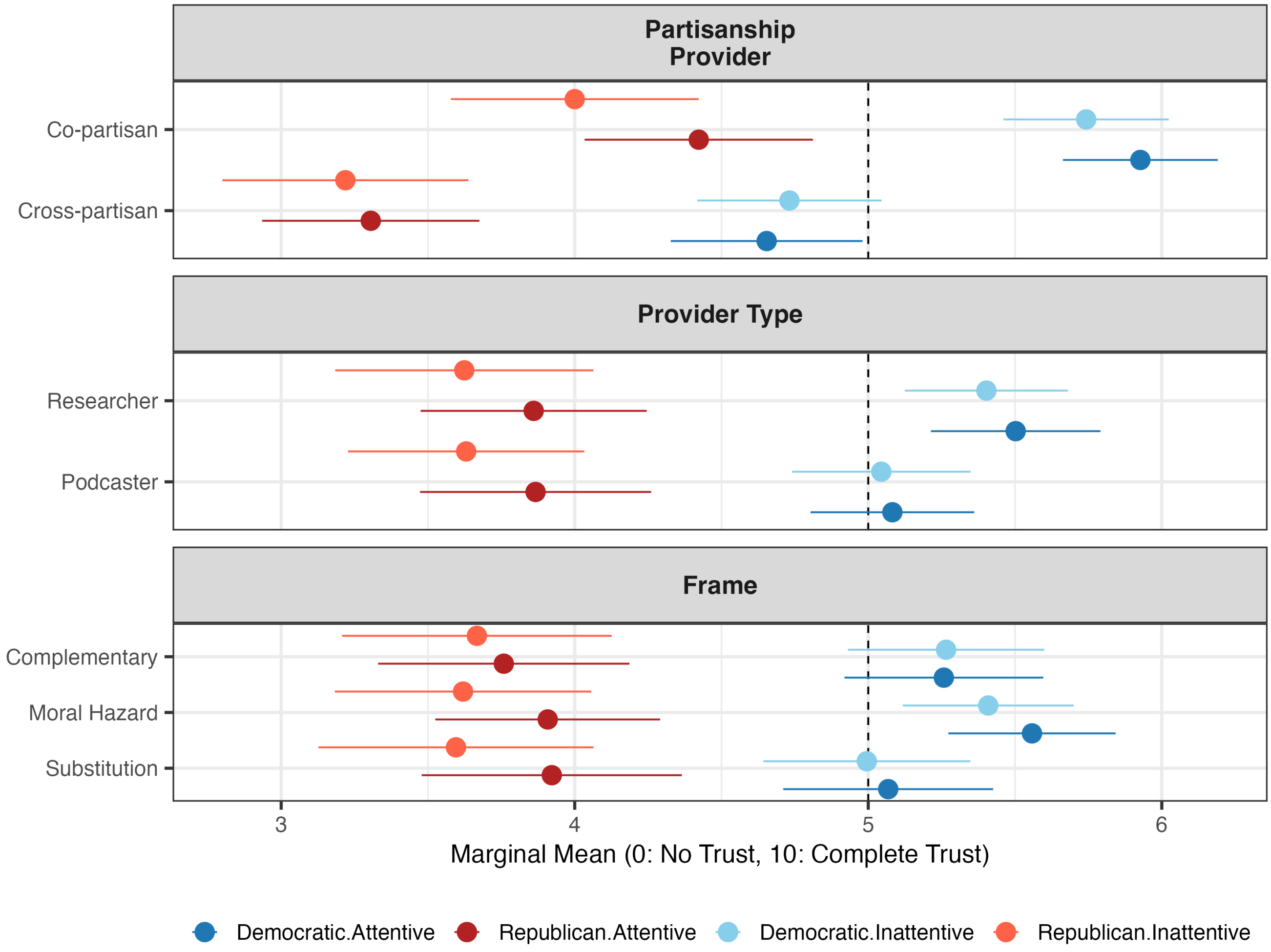

Figure A.13: Marginal means of support for SG by attentiveness and partisanship. Marginal means show the average outcome for each specific level of a conjoint attribute, averaging across all other attributes. Error bars represent 95% confidence intervals.

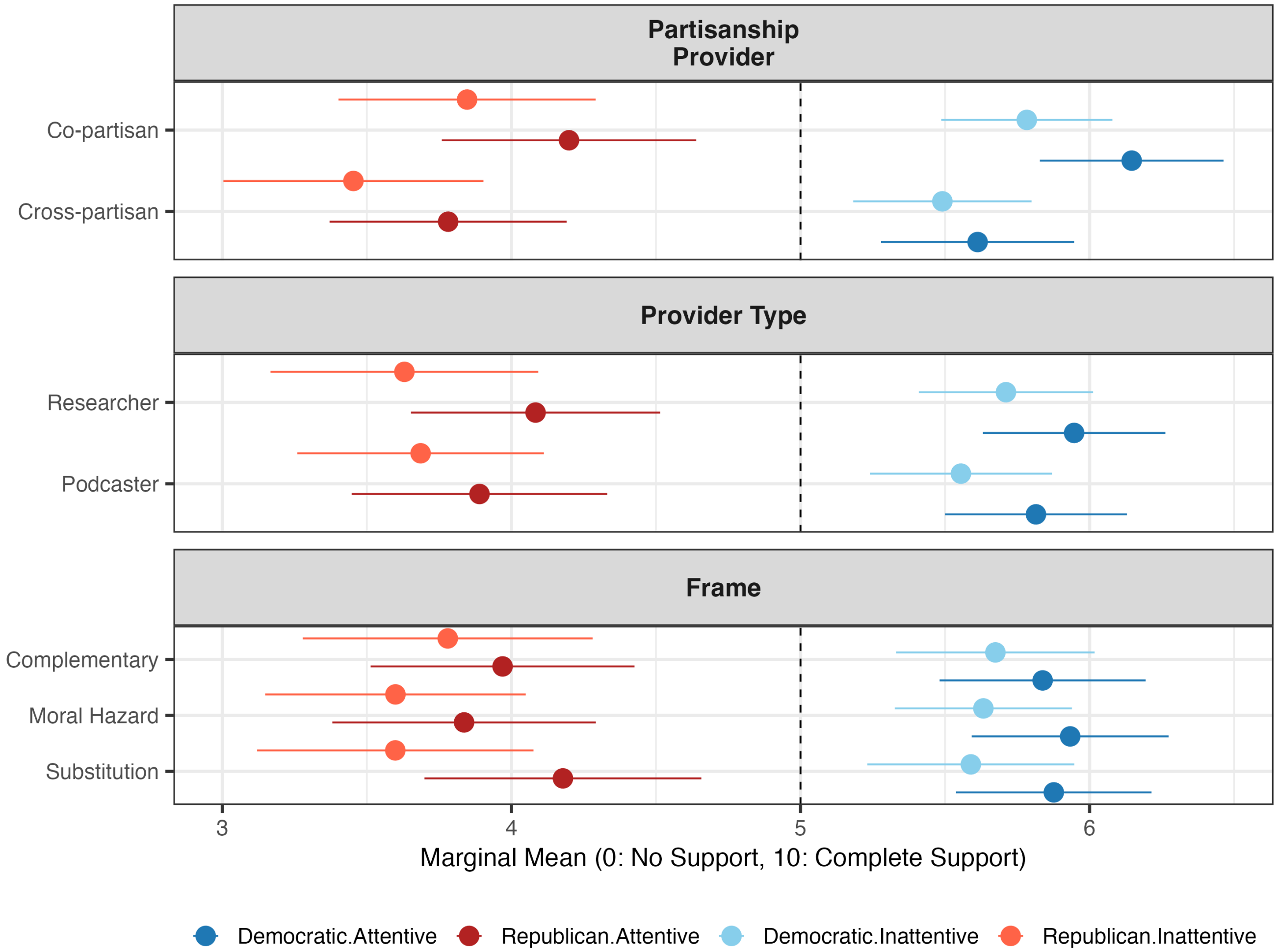

Figure A.14: Marginal means of support for climate mitigation policies by attentiveness and partisanship. Marginal means show the average outcome for each specific level of a conjoint attribute, averaging across all other attributes. Error bars represent 95% confidence intervals.

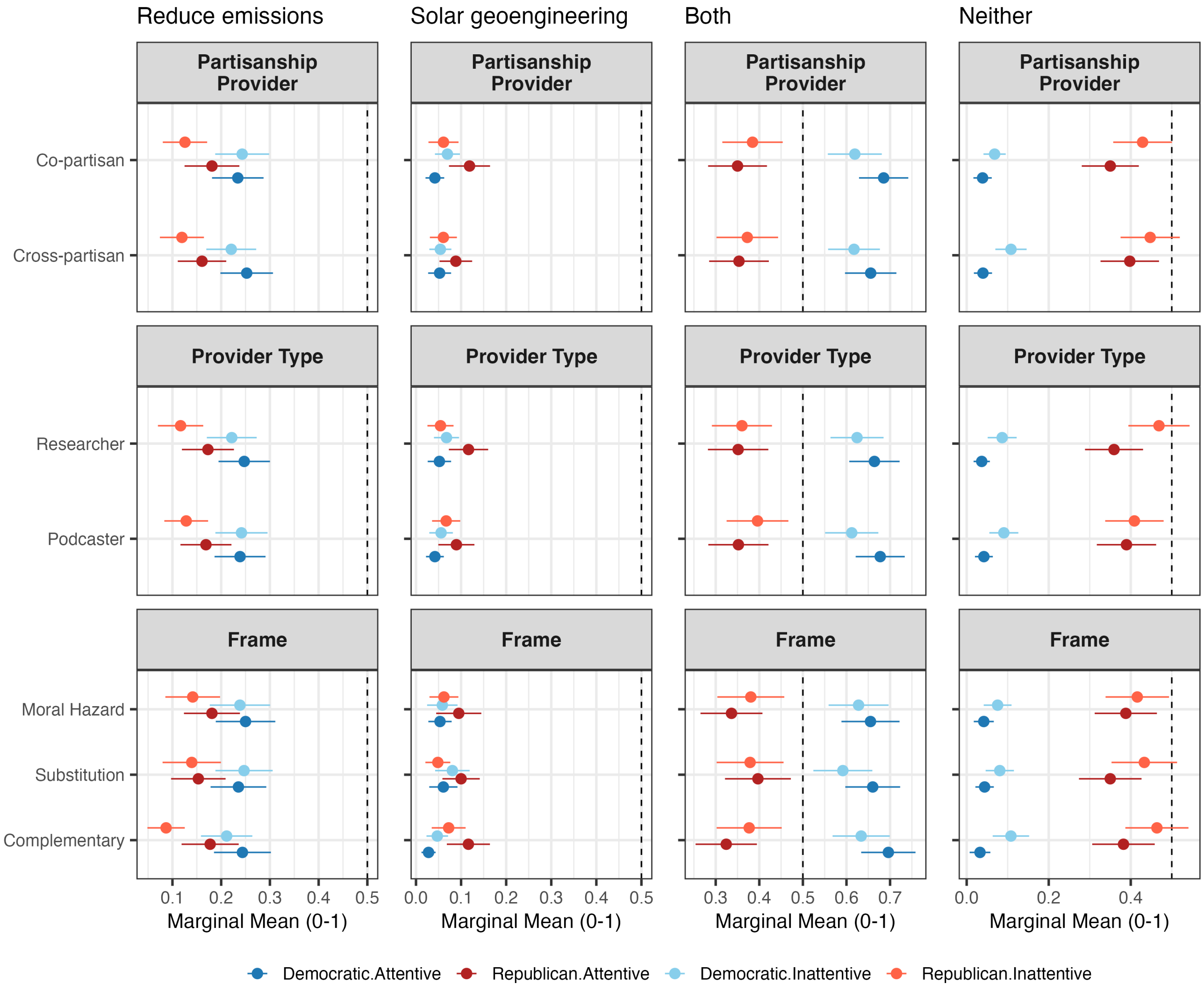

Supplement: Supplementary file 1 — Appendix. [file 44168_2025_236_MOESM1_ESM.pdf]
